# Prevalence and determinants of cigarette smoking among undergraduate medical students in Tanzania: a cross-sectional study

**DOI:** 10.3389/fpubh.2026.1759389

**Published:** 2026-03-19

**Authors:** Zephania Saitabau Abraham, Jackson John Cosmas

**Affiliations:** Department of Surgery, The University of Dodoma, Dodoma, Tanzania

**Keywords:** cigarette, medical students, prevalence, smoking, Tanzania, tobacco, university

## Abstract

**Background:**

Cigarette smoking is a common problem among university students globally and it is one of the leading causes of preventable morbidity and premature mortality globally. It is associated with several risk factors that predispose some individuals to smoke more than others. Medical students play an important role as future health professionals and execute pivotal role models in tobacco control. There has been also a growing burden of smoking-related diseases in Tanzania, not only at the global level, but also among health professionals. However, there is a lack of data on cigarette smoking among university students in Tanzania. This study aimed to determine the prevalence and determinants of cigarette smoking among undergraduate medical students in Tanzania.

**Methods:**

A cross-sectional study was conducted among 410 undergraduate medical students at the University of Dodoma, Tanzania from July to August 2025 using a structured questionnaire. Data analysis was performed descriptively, and chi-square test and logistic regression were conducted using Statistical Package for Social Sciences (SPSS) version 25. Statistical significance was set at 0.05.

**Results:**

A total of 410 students participated in this study and the smoking prevalence was 2.9% (All smokers being men accounting for 100% of smokers). Those living off-campus were significantly more likely to smoke than those on-campus (OR = 12.744; 95% CI = 3.243–50.075; *p* < 0.001), having friends who smoked (OR = 2.326; 95% CI = 0.533–10.146; *p* = 0.261), drinking alcohol (OR = 1.650; 95% CI = 0.306–8.892; *p* = 0.560), being in the fourth year (OR = 1.677; 95% CI = 0.126–22.364; *p* = 0.696), living in urban area (OR = 0.351; 95% CI = 0.066–1.859 *p* = 0.218) and good family relationship (OR = 15.659; 95% CI = 0.557–440.193; *p* = 0.106) were associated with higher odds of smoking though not statistically significant. However, receiving parental advice against cigarette smoking was a significant protective factor (OR = 0.174; *p* = 0.029).

**Conclusion:**

Cigarette smoking is less prevalent among medical students at the University of Dodoma. However, being in the fourth year of study, living off campus, residing in an urban area, consuming alcohol, living with parents, having good family relationships, and having friends who smoke were identified as predictors of cigarette smoking among university students. There is a need to include plans to reduce smoking among university students that can be incorporated into courses or special university programs.

## Introduction

Cigarette smoking is one of the leading causes of preventable morbidity and premature mortality worldwide (1). A smoker is defined as an individual who uses any tobacco product, either daily or occasionally (2). Globally, there are approximately 1.07 billion smokers, including 908 million men and 162 million women, with the majority residing in low- and middle-income countries (3).

According to data from the Global Tobacco Surveillance system, cigarette smoking among men in sub-Saharan Africa ranges from 20 to 60% nationwide, and both men and women are increasingly using tobacco each year (4). In recent years, smoking has become a major contributor to global mortality. By 2020, it was estimated to account for 22.3% of annual deaths worldwide due to excessive tobacco use (5). Cardiovascular diseases, many of which are attributable to cigarette smoking, remain the leading cause of death globally, affecting populations in 182 countries and contributing to 90% of chronic respiratory deaths. Regardless of economic differences, most smokers ultimately die from cardiovascular disease, underscoring the severe impact of tobacco use on one in 10 adults and its detrimental effects on quality of life (6).

Tobacco smoking has numerous detrimental effects on overall health, and it is estimated that smokers die approximately 10 years earlier than non-smokers. Cigarette smoking is associated with an increased risk of lung cancer, chronic obstructive pulmonary disease, atherosclerotic cardiovascular diseases, peptic ulcer disease, intrauterine growth restriction, spontaneous abortion, antepartum hemorrhage, female infertility, male sexual dysfunction, and many other disease conditions (7).

Cigarette smoking is associated with several risk factors that predispose certain individuals to smoke more than others. Studies conducted among young people indicate that social status, level of education, age, and sex play important roles in shaping attitudes toward smoking (8). Various reports also show an increasing number of smokers aged 18 to 22 years, as well as a rise in the proportion of daily smokers who consume more than half a pack of cigarettes per day (9).

Many cigarette smokers initiate the habit between the ages of 13 and 15 years (1). Adolescent smokers are of significant public health concern because they are at increased risk of developing morbidity later in life (10, 11). In 2000, approximately one-third of the global population aged 15 years and older used tobacco, but this declined to 24.9% by 2015. During the same period, the prevalence among males remained three to four times higher than among females (12). In 2016, it was estimated that one-fifth of males and one-third of females worldwide were exposed to second-hand smoke (13).

In Iran, a study conducted to determine the prevalence of cigarette smoking among university students revealed that smoking among male students was 8.9 times higher than among female students, and that recent changes have had no significant impact on smoking prevalence in this group. According to the Iranian study on non-communicable disease risk factors, the prevalence of smoking among men aged 15–64 years is 21.7%, while among those aged 15–24 years it is 7.1%. Therefore, the prevalence of smoking among male university students is 12.5% which is approximately 2.8 times higher than that observed in their counterparts in the general population (14).

A study conducted in Saudi Arabia among university students found that 21.6% had smoked cigarettes. Regarding the age of initiation among current smokers, 17.0% began smoking before the age of 12. The age of initiation was significantly lower among those who smoked only cigarettes compared with those who smoked both cigarettes and waterpipe. Homes and college campuses were the most common places for cigarette smoking (46.0%), whereas waterpipe smokers typically used special venues such as cafés and restaurants (15). Medical students play an important role as future health professionals and execute pivotal role models in tobacco control. There has been also a growing burden of smoking-related diseases in Tanzania, not only at the global level, but also among health professionals. However, there is a lack of data on cigarette smoking among university students in Tanzania. There is a dearth of data on the prevalence of cigarette smoking and its associated factors among undergraduate medical students in Tanzania, and this study aims to address this gap.

## Methods

### Study design, area and study duration

A descriptive cross-sectional study design was used to determine the prevalence and determinants of cigarette smoking among undergraduate medical students at the University of Dodoma. It was conducted from June to August 2025. The university is located at Chimwaga area about eight kilometers east of Dodoma town center and it covers an area of about 15,000 acres. The University of Dodoma comprises of 11 semi-autonomous campuses which include 6 colleges, 3 schools and 2 institutes that have students from both science and art disciplines.

### Study population

The study population consisted exclusively of undergraduate medical students enrolled in the School of Medicine and Dentistry, from the second to the fifth year of their medical training at the University of Dodoma. The total number of second-, third-, fourth-, and fifth-year medical students in the 2024/2025 academic year was 248, 273, 214, and 170, respectively. Among them, males accounted for 193, 218, 171, and 129 students, while females comprised 48, 55, 43, and 41 students in the respective years. A total of 907 medical students were eligible for inclusion in the study.

### Sampling technique

A stratified random sampling technique was employed to select study participants. The study population comprised second- to fifth-year medical students at the University of Dodoma. Due to differences in academic exposure across years of study, stratification by academic year was performed to ensure proportional representation.

The list of all eligible students in each academic year was obtained from the university administration and used as the sampling frame. The total sample size was proportionally allocated to each stratum based on the number of students in that year of study. Within each stratum, students were assigned unique identification numbers, and simple random sampling was performed using a computer-generated random number list to select participants. This procedure ensured that every eligible student had an equal probability of being selected. This approach enhanced the representativeness of the sample and improved the generalizability of the findings to the entire population of second- to fifth-year medical students at the University of Dodoma.

### Operational definitions

Ever smoker: A student who has ever tried smoking cigarettes at any point in his/her lifetime (2).

Current user: A student who has smoked cigarettes on one or more days in the past month (30 days) (2).

Smoker: In this study, a smoker was defined as a participant who had smoked regularly in the 30 days preceding the completion of the questionnaire and had smoked at least 100 cigarettes in their lifetime (16).

Non-smoker: A non-smoker was defined as someone who had not smoked in the previous 30 days and/or had not smoked 100 cigarettes in their lifetime, or who had smoked over 100 cigarettes in their lifetime but none in the last 30 days (16).

### Sample size estimation

The sample size required was calculated by using Kish and Leslie formula.


$$n=[Z^{2}P\;(1-P)]/e^{2}$$

Where;

n is sample size.

Z is standard normal deviation set at 1.96 (corresponding to confidence level of 95%).

P is 60% which is the prevalence of cigarette smoking among university students in Dhaka, Bangladesh (7).

e is marginal error which is tolerated at 5%.

Therefore,


$$n=\frac{1.96\times 1.96\times 60(100-60)}{5\times 5}=369$$

Thus, *n* = 369.

The sample size was estimated to be 369 students.

Adjusting for non-response rate and assuming the non-response rate (f %) to be 10% (17); then the sample size was adjusted upwards to compensate for the expected losses.


$$n^{\prime }=n\;x\;\text{Adjusted factor}$$

Adjusted factor=(100%/100%−f%)

$$n^{\prime }=n\;x\;(100\%/100\%-f\%)$$

$$n^{\prime }=369\;x\;(100\%/100–10\%)\;$$

*n’* = 410

Therefore, the adjusted sample size was 410 undergraduate medical students.

At the time of designing the study, there were no published data on the prevalence of cigarette smoking among medical students in Tanzania. Therefore, we used the most recent and relevant estimate available from Bangladesh as a conservative estimate to ensure adequate sample size. Using a study with a higher prevalence of cigarette smoking ensures sufficient statistical power to detect the existing associations, even if the true prevalence in the population is lower like the established prevalence of cigarette smoking which was estimated to be 3% in our study.

### Inclusion criteria

Undergraduate medical students at the University of Dodoma, from the second to the fifth year of their training, who were willing to participate in the study.

### Exclusion criteria

Students who were unavailable during data collection or who were unwilling to provide consent to participate in the study. First year medical students were excluded since smoking initiation often occurs after prolonged exposure to peers, academic stress, and university social life. First-year medical students may not have had sufficient time for habitual smoking to develop thus their inclusion could have underestimated the prevalence of cigarette smoking linked to university-specific factors.

### Data collection tools

In this study the primary data collection tool was through a standard semi-structured English electronic questionnaire. The data collection instrument has been adapted from validated tools in studies published elsewhere (1–3, 7, 18). Since university medical students are well fluent in terms of reading and understanding English, the tool was developed in English language.

The questionnaire consisted of open and close-ended questions being prepared by the use of kobo toolbox.

The questionnaire consisted of 4 sections; Section (1) comprised socio-demographic characteristics such as age, gender, year of study; Section (2) characterized cigarette smoking status for example current smoking status, frequency, and quantity; Section three (3) determined factors or predictors of cigarette smoking such as peer pressure, stress, socioeconomic status, parental smoking, and accessibility to cigarettes and section (4) determined health related problems of cigarette smoking.

In this study, the primary data collection tool was a standard structured electronic questionnaire in English. The questionnaire included closed-ended questions and was developed using Kobo Toolbox. It comprised four sections: Section 1 covered socio-demographic characteristics such as age, gender, and year of study. Section 2 assessed cigarette smoking status, including current smoking, frequency, and quantity. Section 3 examined determinants or predictors of cigarette smoking, such as peer pressure, stress, socioeconomic status, parental smoking, and access to cigarettes. Section 4 focused on health-related problems associated with cigarette smoking.

### Measurement of variables

#### Dependent variable

The dependent variable in this study was cigarette smoking status, categorized as current smoker, non-smoker, or ex-smoker.

#### Independent variables

The independent variables included socio-demographic characteristics (age, gender, year of study, parental smoking); environmental factors (peer influence, availability of cigarettes); and psychological factors (stress levels and academic pressure).

### Reliability and validity

Reliability was assessed using the test–retest method in which 20 medical students in their second and third years of training from the School of Medicine and Dentistry who were not involved in the study completed the final English version of the questionnaire twice within 2 weeks. Outcomes of the two times were compared using Pearson’s correlation coefficient (Pearson’s r) as a reliability test. A more significant stability coefficient (Pearson’s r) suggested a good test retest reliability.

### Internal consistency

Regarding internal consistency between items in the survey, it was measured using the coefficient alpha “Cronbach’s alpha.” A Cronbach *α* = 0.785 was obtained, suggesting adequate internal consistency.

### Content validity

Content validity was assessed by distributing this modified English version questionnaire among the expert panel belonging to the specialty of public health as well as Otorhinolaryngology. Rating was done based on relevance, clarity, simplicity, and ambiguity.

### Data processing and analysis

Data were collected using a structured electronic questionnaire and analyzed using SPSS version 25. Demographic characteristics were analyzed using descriptive statistics to present frequencies and percentages. Logistic regression analyses (both univariate and multivariate) were conducted to determine the relationships between variables associated with cigarette smoking. An independent variable with a *p*-value of less than 0.05 was considered statistically significant.

### Ethical approval and consent to participate

Ethical clearance was obtained from the Institutional Research Review Ethics Committee (IRREC) of the University of Dodoma (Ref. No. MA.84/261/93/67). Permission to conduct the study was granted by the University of Dodoma, School of Medicine and Dentistry, in August 2025. Before recruitment, all potential participants were required to provide informed consent by signing a consent form after receiving detailed information about the study’s purpose and significance. They were informed that participation was entirely voluntary and that they were free to decide whether to participate, provided they met the inclusion criteria.

All data collected in this study were treated with strict confidentiality. Participants’ names did not appear on the questionnaires, as coding was used to maintain anonymity. Additionally, no information obtained from participants was shared with individuals outside the research team. All data remained confidential and were used solely for the purposes of this study.

## Results

### Socio-demographic characteristics among undergraduate medical students at the University of Dodoma

A total of 410 students participated in the study. The majority were aged 24–27 years (62.4%). Males (79.5%) predominated in this study and nearly all participants were single (98.3%). Most were in their fourth (31.2%) or fifth year of study (31.7%). The majority resided on-campus (91.5%), had an urban permanent residence (62.7%), and were living with their parents (93.7%) (Table 1).

**Table 1:** Socio-demographic characteristics of undergraduate medical students.

Variable	Characteristics	Frequency, n(%)
Age (years)	20–23	138 (33.7)
	24–27	256 (62.4)
	28 and above	16 (3.9)
Sex	Male	326 (79.5)
	Female	84 (20.5)
Marital status	Married	7 (1.7)
	Single	403 (98.3)
Year of study	Second year	101 (24.6)
	Third year	51 (12.4)
	Fourth year	128 (31.2)
	Fifth year	130 (31.7)
Residence at the university	On-campus	375 (91.5)
	Off-campus	35 (8.5)
Place of permanent residence	Urban	257 (62.7)
	Rural	153 (37.3)
Living with parents	Yes	384 (93.7)
	No	25 (6.1)
Father’s education level	College/University	225 (54.9)
	Neither read nor write	1 (O.2)
	Primary school	64 (15.6)
	Secondary school	120 (29.3)
Mother’s education level	College/University	76 (18.5)
	Neither read nor write	1 (0.2)
	Primary school	93 (22.7)
	Secondary school	240 (58.5)
Main source of income while at university	Government sponsorship	313 (76.3)
	Parents/family members	44 (10.7)
	Self-pocket	52 (12.7)
	Faith- based sponsorship	1 (0.2)

### Prevalence and patterns of cigarette smoking among undergraduate medical students

Cigarette smoking was uncommon among participants, with only 2.9% reporting ever smoking. Among these, the majority were current smokers (91.7%), predominantly occasional users (72.7%). Most smokers initiated smoking during college or university (72.7%) and reported consuming fewer than three cigarettes per day (63.6%) (Table 2).

**Table 2:** Prevalence and patterns of cigarette smoking among undergraduate medical students.

Variables	Characteristics	Frequency n(%)
Have you ever smoked cigarette	Yes	12 (2.9)
	No	398 (97.1)
Are you currently smoking (*n* = 12)	Yes	11 (91.7)
	No	1 (8.3)
How often do you smoke (*n* = 11)	Occasionally	8 (72.7)
	Weekly	3 (27.3)
When did you start cigarette smoking (*n* = 11)	During college/University education	8 (72.7)
	During secondary education	3 (27.3)
How many cigarettes do you smoke per day (*n* = 11)	<3	7 (63.6)
	3 and above	4 (36.4)

### Health-related problems of cigarette smoking among undergraduate medical students

All smokers expressed concern about their smoking habits, and the majority reported attempting to quit (72.7%). Behavioral changes were the most commonly perceived challenge to cessation (81.8%). Few smokers (18.2%) reported smoking-related health problems, and none had sought treatment (Table 3).

**Table 3:** Health related problems of cigarette smoking among undergraduate medical students.

Variables	Characteristics	Frequency n(%)
Ever worried about cigarette smoking (*n* = 11)	Yes	11 (100)
	No	0(0)
Are you trying to quit (*n* = 11)	Yes	8 (72.7)
	No	3 (27.3)
Which health problems may occur when you quit smoking (*n* = 11)	Behavioral change	9 (81.8)
	Missing class	2 (18.2)
Do you have any health problem related to cigarette smoking (*n* = 11)	Yes	2 (18.2)
	No	9 (81.8)
Was treatment provided for the health problem you encountered related to smoking (*n* = 2)	Yes	0 (0)
	No	2 (100)

### Determinants or predictors of cigarette smoking among undergraduate medical students

In multivariable analysis, residing off-campus was strongly associated with smoking (OR = 12.74; *p* < 0.001). Conversely, receiving parental advice against smoking was a significant protective factor (OR = 0.17; *p* = 0.029). Other factors, including having friends who smoked, alcohol consumption among students, academic year, and family relationship status, were not significantly associated with smoking (Table 4).

**Table 4:** Determinants or predictors of cigarette smoking among undergraduate medical students.

Variables	Characteristics	Smoking status		Multivariable logistic regression model			
		Smokers n(%)	Non-smokers n(%)	OR(95%CI)	SE	*β*	*p*
Age (years)	20–23	2 (18.2)	136 (34.1)	0.000	8184.103	−16.549	0.998
	24–27	9 (81.8)	247 (61.9)	0.000	8184.103	−17.516	0.998
	28 and above	0 (0.0)	16 (4.0)	Ref			
Sex	Male	12 (100.0)	315 (78.9)	0.000	4015.488	−17.882	0.996
	Female	0 (0.0)	84 (21.1)	Ref			
Marital status	Married	0 (0.0)	7 (1.7)	Ref			
	Single	11 (100.0)	392 (98.3)	0.000	11888.842	−15.086	0.999
Year of study	Second year	1 (9.1)	100 (99.0)	0.714 (0.016–30.906)	1.923	−0.337	0.861
	Third year	1 (9.1)	50 (98.0)	Ref			
	Fourth year	3 (27.3)	125 (97.6)	1.677 (0.126–22.364)	1.322	0.517	0.696
	Fifth year	6 (54.5)	124 (95.4)	0.768 (0.065–9.083)	1.260	−0.263	0.834
Residence at the university	On-campus	4 (36.4)	371 (98.9)	Ref			
	Off-campus	7 (63.6)	28 (80)	12.744 (3.243–50.075)	0.698	2.545	0.000
Place of permanent residence	Urban	8 (72.7)	249 (96.9)	0.351 (0.066–1.859)	0.850	−1.047	0.218
	Rural	3 (27.3)	150 (98.0)	Ref			
Living with parents	Yes	10 (90.9)	375 (97.4)	0.109 (0.000–34.291)	2.933	−2.213	0.450
	No	1 (9.1)	25 (96.2)	Ref			
Relationship with your family	Good	8 (72.7)	397 (98.0)	15.659 (0.557–440.193)	1.702	2.751	0.106
	Not good	3 (27.3)	2 (66.7)	Ref			
Smokers among close/friend/peer/peer pressure	Some of them	7 (63.6)		2.326 (0.533–10.146)	0.751	0.844	0.261
	None	4 (36.4)		Ref			
Do you drink alcohol	Yes	6 (54.5)		1.650 (0.306–8.892)	0.859	0.501	0.560
	No	5 (45.5)		Ref			
Ever received parental advice against smoking	Yes	4 (36.4)		0.174 (0.036–0.838)	0.801	−1.748	0.029
	No	7 (63.6)		Ref			

## Discussion

This study was conducted to determine the prevalence and determinants of cigarette smoking among undergraduate medical students at the University of Dodoma. The findings provide important insights into the extent of smoking and the factors associated with it among the university students.

This study found that the prevalence of cigarette smoking among the participants was 2.9%, which is lower than reported in studies of university students in other countries. For example, a study in Ethiopia reported a prevalence of 6.8% among college students (2), while a study in Bangladesh found a prevalence of 60% among university students (7) and the study among dental students in Tanzania found the prevalence of cigarette smoking to be 12.8% (19). The relatively low prevalence of cigarette smoking in this study may be due to campus smoking-free policies, and sociocultural norms among medical students where they are socialized into a professional culture that promotes healthy behaviors. Smoking may be perceived as inconsistent with the role of a future healthcare provider and the expectation to model healthy lifestyle can discourage tobacco use (20). The low prevalence of cigarette smoking may thus be because of underreporting due to social desirability.

Most smokers in this study began cigarette smoking during their university years (72.7%). This finding is consistent with studies conducted at Tuzla University in Albania (18) and in Bangladesh (7), which reported that cigarette smoking commonly begins in late adolescence or early adulthood. Reduced monitoring due to lack of parental supervision while at the university may increase experimentation with risk behaviors, including cigarette smoking and at times smoking may be initiated as a perceived coping mechanism for stress.

In this study, cigarette smoking was mostly occasional (72.7%) and light, with most students smoking fewer than three cigarettes per day. This contrasts with findings from Tuzla University in Albania, where students smoked an average of 15 cigarettes daily (18).

The predominance of occasional and light smoking in this study may reflect strong socio-cultural norms, heightened health awareness among medical students, and possible economic constraints limiting cigarette smoking. Additionally, smoking may represent early-stage experimentation rather than established nicotine dependence thus cigarette smoking being on light basis in early stages of smoking during university life. These contextual factors may explain the contrast with findings from Tuzla University, where students reported substantially higher daily cigarette consumption.

In this study, peer influence emerged as an important factor in the initiation of cigarette smoking, with 63.6% of smokers reporting that some of their friends smoked. This finding aligns with evidence from a study in Bangladesh, where over 62% of students reported starting smoking due to peer influence (7). In contrast, exposure to family members who smoke has been documented as a strong predictor in other studies. For example, a study in Ethiopia found that students with family members who smoked were six times more likely to smoke compared to those with no such exposure (2). Similarly, in Bangladesh, 62% of students reported being influenced by friends and also by imitation of family members (7). This differs from our study, where only 9.1% of smokers reported being influenced by family members who smoke.

This study also identified living off-campus as a significant predictor of cigarette smoking, with 63.6% of smokers reporting off-campus residence. Students living off-campus had 12.7 times higher odds of smoking compared to those living on-campus. This finding contrasts with a study in Ethiopia, which reported that students residing in hostels were 11.62 times more likely to smoke compared to those living at home (2). The difference may be attributed to the strict restrictions and anti-smoking policies enforced within and around the University of Dodoma campus.

Also cigarette smoking was more common among male students (100%), this pattern is similar to the study that was done in Iran to determine the prevalence of cigarette smoking among students in Iran’s universities which reported that smoking among male students was 8.9 times higher than among females (14) and also similar to the study done in Bangladesh, where male prevalence was significantly higher (7). In many Asian and African societies, smoking is more socially acceptable for men than for women. Female smoking may be stigmatized, viewed as culturally inappropriate, or associated with negative moral perceptions. As a result, women may be less likely to initiate smoking. On top of this, male students often experience greater social freedom, including participation in social gatherings like clubs where smoking and alcohol use are more common. This increased exposure may raise the likelihood of smoking initiation in males than female medical students.

All smokers in this study expressed concern about their smoking habits, and most (72.7%) were attempting to quit. This finding is consistent with a study at Tuzla University in Albania, where more than half of the respondents (56.1%) were trying to quit smoking (18). As medical students are likely to have strong knowledge of the adverse health effects of cigarette smoking, including cancer, cardiovascular disease, and respiratory illness, this heightened awareness may increase guilty, concern, and motivation to quit from smoking. This may also be coupled with strong anti-cigarette smoking policies at the University.

Additionally, most participants in this study (81.8%) identified behavioral changes as a challenge when attempting to quit. This contrasts with the Tuzla University study, where 24.1% of participants reported stress or boredom as potential challenges to quitting (18). Given that most participants in this study were light and occasional smokers, behavioral routines and environmental triggers may pose greater challenges than physiological withdrawal. Differences in academic environment and socio-cultural context may explain the contrast with findings from Tuzla University, where stress and boredom were more commonly reported challenges when attempting to quit from cigarette smoking.

## Conclusion

Cigarette smoking was infrequent among medical students at the University of Dodoma. Notably, all smokers expressed concern about their habit and majority reported attempts to quit. However, the very small number of smokers identified in this study limits the robustness of conclusion regarding determinants of smoking and may have reduced the stability of regression estimates. Further studies with larger samples of cigarette smokers are recommended to better understand the predictors of cigarette use in this important population. Strengthening the existing university cigarette smoking measures and health promotion initiatives may help sustain the low prevalence observed.

### Study limitations and strength

This study has several limitations, including its cross-sectional design, small sample size, and being conducted at a single educational institution in Tanzania, which limits the generalizability of the findings to all medical students nationwide. Additionally, selection bias may further restrict the applicability of the results. The relatively small number of smokers identified in this study may have limited the adequacy of the sample size for multivariable logistic regression analysis. This could have reduced the statistical power and affected the stability of the estimated associations. Therefore, the findings from the regression analysis should be interpreted with caution. Nevertheless, given the limited data on cigarette smoking among university students in developing countries, particularly in Tanzania, this study provides valuable insights that contribute to the existing literature.

## Data Availability

The raw data supporting the conclusions of this article will be made available by the authors, without undue reservation.
